# Comparative Evaluation of Tooth Discoloration Induced by an Experimental Antibiotic Paste Modified with Nano Chitosan: An In Vitro Study

**DOI:** 10.3390/dj13070307

**Published:** 2025-07-09

**Authors:** Mohamed Ahmed Elsayed, Md Sofiqul Islam, Safiya Ali, Zainab Hussain, Muhammed Mustahsen Rahman, Okba Mahmoud

**Affiliations:** 1 Department of Endodontics, RAK College of Dental Sciences, RAK Medical and Health Sciences University, Ras Al-Khaimah P.O. Box 12973, United Arab Emirates; mohamed.elsayed@rakmhsu.ac.ae; 2 Department of Endodontics, Faculty of Dentistry, Assiut University, Assiut 71516, Egypt; 3 Department of Operative Dentistry, RAK College of Dental Sciences, RAK Medical and Health Sciences University, Ras Al-Khaimah P.O. Box 12973, United Arab Emirates; sofiqul.islam@rakmhsu.ac.ae; 4 Department of Clinical Science, RAK College of Dental Sciences, RAK Medical and Health Sciences University, Ras Al-Khaimah P.O. Box 12973, United Arab Emirates; safiyaalarayedh36@gmail.com (S.A.); zainabah.20011@gmail.com (Z.H.); 5 Department of Periodontology, RAK College of Dental Sciences, RAK Medical and Health Sciences University, Ras Al-Khaimah, P.O. Box 12973, United Arab Emirates; mustahsen@rakmhsu.ac.ae; 6 Department of Clinical Sciences, College of Dentistry, Ajman University, Ajman P.O. Box 346, United Arab Emirates; 7 Center for Medical and Bio-Allied Health Sciences Research, Ajman University, Ajman P.O. Box 346, United Arab Emirates

**Keywords:** calcium hydroxide, intracanal medicaments, nano chitosan, spectrophotometric analysis, triple antibiotic paste, tooth discoloration

## Abstract

**Background/Objectives:** Tooth discoloration is a common concern associated with the use of intra-canal medicaments, particularly those containing antibiotics. This study aims to evaluate the tooth discoloration potential of an experimental antibiotic paste modified with Nano Chitosan (APNC) and compare it with two antibiotic pastes and two calcium hydroxide-based pastes over different time intervals. **Methods**: Fifty bovine incisors were standardized and prepared up to size 60. The teeth were randomly assigned into five groups based on the medicament applied: Metapaste, Metapex, modified Triple Antibiotic Paste (mTAP), Double Antibiotic Paste (DAP), and APNC. A digital spectrophotometer was used to measure the color parameters (L*, a*, and b*) at two zones, above and below the cemento-enamel junction (CEJ), across four-time points: before application (T0- baseline), immediately after application (T1), after two weeks (T2), and after one month (T3). The color changes (ΔE) were calculated and statistically analyzed using repeated-measure ANOVA. **Results**: Statistically significant differences in discoloration were observed between the tested medicaments after one month (*p* < 0.05). mTAP caused the highest ΔE values both above and below the CEJ at all time points, particularly after one month (*p* < 0.05). Conversely, APNC, Metapaste, and DAP demonstrated the least discoloration, with no significant differences among them. The degree of discoloration was time-dependent and more pronounced below the CEJ in all groups. **Conclusions**: After one month, the experimental APNC paste induced tooth discoloration comparable to that of Metapaste, indicating minimal esthetic compromise. APNC may be a promising alternative to traditional antibiotic pastes with minimal discoloration effects.

## 1. Introduction

Achieving successful root canal treatment (RCT) requires the thorough elimination of bacteria, biofilm, and necrotic tissue from the root canal system, which is particularly challenging in heavily infected cases with necrotic pulp. Disinfection strategies typically involve mechanical debridement using different endodontic files and chemical disinfection with potent antimicrobial agents such as sodium hypochlorite (NaOCl). However, in cases of persistent infection, particularly those associated with large periapical radiolucencies, intracanal medicaments are recommended to further disinfect the canal before obturation [1].

One of the most commonly used intracanal medicaments is calcium hydroxide (Ca(OH)_2_), which exhibits strong antimicrobial activity, an ability to dissolve necrotic tissue, and low toxicity [2]. Despite these advantages, calcium hydroxide has several limitations, including incomplete eradication of complex bacterial flora and biofilm in cases of persistent infection, and negatively affects dentin microhardness when used for prolonged periods, leading to increased fracture susceptibility [3]. To overcome the limitations of calcium hydroxide, Triple Antibiotic Paste (TAP) has been introduced as an alternative intracanal disinfectant [4]. TAP contains a combination of ciprofloxacin, metronidazole, and minocycline, which together provide broad-spectrum antimicrobial coverage against Gram-positive and Gram-negative bacteria, as well as aerobic and anaerobic bacteria commonly found in infected root canals [5].

Ciprofloxacin, a fluoroquinolone, disrupts bacterial DNA replication by inhibiting DNA gyrase, making it particularly effective against Gram-negative bacteria. Metronidazole targets anaerobic bacteria by interfering with DNA synthesis, while minocycline, a tetracycline-class antibiotic, inhibits protein synthesis in the bacterial cells by binding to the 30S ribosome, demonstrating strong activity against Gram-positive organisms [6]. While TAP is highly effective for bacterial eradication, concerns have been raised regarding its cytotoxic effects on dental pulp stem cells, particularly when used at high concentrations [7]. Additionally, the most significant drawback of TAP is tooth discoloration, primarily due to minocycline, which binds to dentin collagen and induces intrinsic tooth staining that can be difficult to reverse [8]. This discoloration is particularly problematic in anterior teeth, where esthetics play a crucial role in patient satisfaction [9]. To mitigate this issue, modified formulations of TAP have been developed. These include Modified Triple Antibiotic Paste (mTAP), in which minocycline is replaced with alternative antibiotics such as clindamycin or cefaclor, as well as Double Antibiotic Paste (DAP), which contains only metronidazole and ciprofloxacin, thereby eliminating the discoloration associated with minocycline [10]. While these alternative formulations maintain antimicrobial efficacy, the extent of their impact on color stability remains an important clinical consideration as the discoloration potential and its effect on the hue, chroma, and value of teeth are unique for each antibiotic [11]. The current guidelines recommend the use of different alternative medications to avoid this discoloration effect [12].

In recent years, the introduction of nano-based intracanal medicaments has emerged as a promising strategy to improve antimicrobial effectiveness while minimizing undesirable side effects. Nanoparticles exhibit superior antibacterial properties due to their high surface area-to-volume ratio, allowing them to penetrate biofilms and dentinal tubules more effectively than traditional formulations, and demonstrate sustained antimicrobial activity within the root canal system [13]. Among these, Nano Chitosan has gained significant attention due to its biocompatibility, antimicrobial activity, and dentin-penetrating capabilities [14]. Chitosan is a naturally occurring polysaccharide derived from the deacetylation of chitin, found in crustacean shells. Its nanoparticles (Chitosan Nanoparticles, CNPs) not only exhibit potent antibacterial effects but also possess chelation properties that may reduce biofilm formation and enhance tissue regeneration [15]. Preliminary studies suggest that Nano Chitosan-modified antibiotic pastes may offer several advantages over traditional TAP and DAP, including enhanced biofilm disruption, prolonged antimicrobial activity, and potentially lower discoloration effects [16]. However, despite these advantages, the discoloration potential of Nano Chitosan-based medicaments has not been fully investigated, making it imperative to evaluate their esthetic impact before widespread clinical adoption. Given the critical role of esthetics in endodontic therapy, especially in young adult patients, it is essential to assess the color stability of these novel formulations.

The findings of this study will provide insight into the esthetic outcomes of intracanal medicaments, particularly evaluating whether Nano Chitosan-based formulations such as APNC can serve as effective alternatives with minimal discoloration, which is especially relevant when treating infected canals in anterior zone where esthetic preservation remains a significant concern. This study aims to compare the color changes (ΔE) induced by an experimental antibiotic paste modified with Nano Chitosan (APNC) against commonly used intracanal medicaments such as Metapaste, Metapex, modified Triple Antibiotic Paste (mTAP), and Double Antibiotic Paste (DAP), over various time intervals. The following null hypotheses were formulated: (i) there is no significant difference in the degree of tooth discoloration induced by the tested medicaments after one month; (ii) all medicaments produce a similar degree of discoloration in both zones—above and below the cemento-enamel junction (CEJ); and (iii) all medicaments exhibit a similar progression of discoloration across the three post-application time points.

## 2. Materials and Methods

### 2.1. Sample Size Calculation and Selection

A power analysis was conducted to ensure the study had sufficient statistical power to detect significant differences in tooth discoloration among the tested groups. The analysis aimed for a statistical power of 80% (β = 0.2), with an alpha level of 0.05. Using one-way ANOVA power analysis (SPSS 26), and assuming a mean standard deviation of 1.89 and an effect size of 0.487, obtained from previous similar studies [8,11], the required sample size was calculated to be 10 teeth per group. Fifty freshly extracted bovine incisors from animals aged between 3 and 5 years were collected following ethical approval obtained from the Institutional Ethical Committee (RAKMHSU-REC-012-2022/23-UG-D). Immediately after extraction, the teeth were disinfected by immersion in 0.5% Chloramine-T solution and stored in 0.9% sterile saline solution with neutral pH and refrigerated at 5 °C to prevent dehydration and preserve structural integrity. Soft tissue remnants and surface debris were meticulously removed using a scalpel. Further cleaning was performed using an ultrasonic scaler to eliminate residual organic and mineral deposits. Each tooth was examined under a dental operating microscope (CJ-Optik GmbH, Asslar-Werdorf, Germany,) at 20× magnification to detect any visible cracks, defects, or structural anomalies that could compromise the study’s validity. Only intact teeth with similar dimensions were selected to ensure standardization and homogeneity across all experimental groups.

### 2.2. Sample Preparation

**Figure 1:**
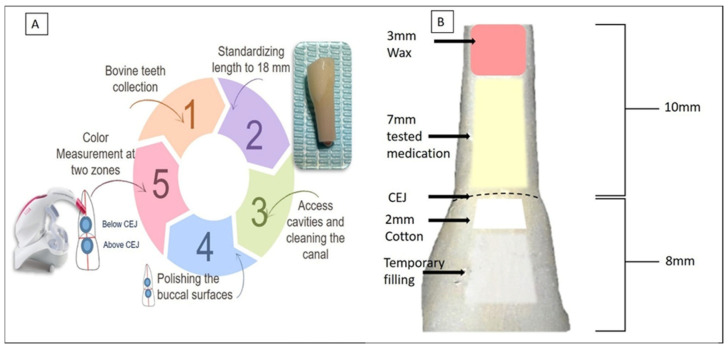
(**A**) Schematic illustration of the experimental procedure. (**B**) Standardization of tooth length to 18 mm, comprising 10 mm of root and 8 mm of crown. The root apices were first sealed with a 3 mm wax plug. Intracanal medicaments were injected up to the cemento-enamel junction (CEJ) level. A 2 mm cotton pellet was placed above the CEJ, and access cavities were sealed with a temporary filling.

The experimental procedure is schematically presented in Figure 1A. To standardize tooth length, the apical third and incisal edges of each specimen were trimmed using a diamond disc under copious water irrigation, ensuring a final tooth length of 18 mm. The crown-to-root ratio was maintained at a consistent 8 mm from the crown to the CEJ and 10 mm from the CEJ to the root apex (Figure 1B). A standardized access cavity was created in each tooth using a high-speed diamond bur under continuous water spray. Pulp tissue was removed using K-files (#40 to #60, Dentsply Maillefer, Ballaigues, Switzerland). During instrumentation, the pulp chamber and root canal were irrigated with 5 mL of 5.25% sodium hypochlorite (NaOCl) solution (Chloraxid, Cerkamed, Stalowa Wola, Poland). After completion of instrumentation, a final irrigation protocol was performed using 1 mL of 17% ethylenediaminetetraacetic acid (EDTA) (META Biomed Co., Ltd., Daejeon, Republic of Korea) for one minute, followed by 5 mL of distilled water to remove any residual irrigants. The pulp chambers and root canal space were then dried using cotton, and the canals were thoroughly dried using sterile paper points. Finally, the apex was sealed with a 3 mm wax plug (Figure 1B).

### 2.3. Baseline Color Measurements

To ensure standardized and reproducible color measurements, two circular adhesive strips with a diameter of 6 mm were affixed to the buccal surfaces of each tooth, one positioned above the CEJ and the other below it. These designated zones provided consistent reference points for spectrophotometric assessment. The surfaces within the adhesive strips were polished using 1500-grit silicon carbide paper to achieve a flat, smooth, and uniform area, thereby minimizing variability due to surface curvature. Following surface preparation, the specimens were randomly assigned into five experimental groups (*n* = 10 per group) using a manual randomizer based on the intracanal medicament applied: Group 1 (Metapaste), a calcium hydroxide paste (Meta Biomed, Daejeon, Republic of Korea); Group 2 (Metapex), a calcium hydroxide paste containing iodoform (Meta Biomed, Republic of Korea); Group 3 (mTAP), a modified Triple Antibiotic Paste; Group 4 (DAP), a Double Antibiotic Paste; and Group 5 (APNC), an antibiotic paste with Nano Chitosan. Each tooth was assigned a numerical code without any identifying data to ensure blinded analysis.

To record the baseline color parameters (T0, before the application of the medicaments), a digital spectrophotometer, VITA Easyshade^®^ Advance 4.0 (VITA Zahnfabrik, Bad Säckingen, Germany), was used under standardized lighting conditions. The device was calibrated prior to each measurement session, and the measuring probe was placed perpendicular and flush to the tooth surface. All measurements were performed against a white background to minimize external light interference following the guideline given by Islam MS et al. [17]. Color data were recorded based on the Commission Internationale de l’Eclairage (CIE) Lab* color space system, which defines three parameters: L* (lightness), a* (red-green axis), and b* (yellow-blue axis). For each zone (above and below the CEJ), three readings were taken per specimen, and the mean value was recorded.

### 2.4. Preparation of Tested Materials

The composition of tested materials is presented in Table 1. Three experimental groups (mTAP, DAP, and APNC) included antibiotic-based pastes prepared immediately before application to ensure consistency. For Group 3, the mTAP was prepared by mixing 500 mg of each of the following antibiotic powders in a 1:1:1 ratio: Amoxicillin + clavulanate (Augmentin, 1.2 g vial, GlaxoSmithKline, Cairo, Egypt), ciprofloxacin (Ciprocin 500 mg, EPICO, Cairo, Egypt), and doxycycline (Vibramycin 100 mg, Pfizer, Cairo, Egypt). The powders were blended thoroughly using a mortar and pestle to ensure a homogeneous mixture. For Group 4, the DAP was prepared by mixing equal portions (1:1 ratio) of metronidazole (Flagyl 500 mg, Aventis, Cairo, Egypt) and ciprofloxacin. For Group 5, the APNC consisted of a 1:1:1 mixture of ciprofloxacin, Amoxicillin + clavulanate, and 500 mg of chitosan nanopowder (Sisco Research Laboratories Pvt. Ltd., Mumbai, India). The chitosan nano-powder used had an assay of 99% purity and an average particle size of 80–100 nm. The powders were thoroughly mixed to ensure even dispersion of nanoparticles within the antibiotic matrix. Immediately before use, the powdered components of Groups 3, 4, and 5 were mixed with sterile distilled water at a powder-to-liquid ratio of 3:1. Approximately 1–2 mL of water was gradually added while triturating with a pestle until a smooth, creamy, paste-like consistency was achieved. The final paste was adjusted to be cohesive enough without being too dry or too watery.

### 2.5. Application of Tested Materials

The intracanal medicaments were applied as follows: Groups 1 (Metapaste) and Group 2 (Metapex) were injected into the canals using the manufacturer-provided intracanal delivery tips. The injection depth was standardized at 5 mm apical to the CEJ, and the volume of paste was controlled using 2 reference markings on the dispensing tubes. In Groups 3 (mTAP), 4 (DAP), and 5 (APNC), the freshly prepared experimental pastes were delivered into the canals using sterile plastic syringes fitted with 20-gauge needles. A standardized volume of 0.3 mL of each medicament was carefully injected into the canal. Care was taken to minimize the presence of voids or gaps within the canal. Following medicament placement, a premeasured 2 mm thick sterile cotton pellet was placed at the level of the CEJ in each tooth. The access cavities were then sealed with a temporary restorative material (Cavit, 3M ESPE, Neuss, Germany) to prevent external contamination and dehydration of the samples (Figure 1B). All steps, including the removal of the pulp tissue, irrigation, and placement of intracanal medicaments, were performed through the coronal access by the same operator.

#### Color Measurement over Observational Time Points

Color parameters (L*, a*, and b*) were recorded for each sample immediately after medicament application (T1). The specimens were stored in sterile 0.9% saline solution in sealed containers at 100% humidity and 37 °C in an incubator between color measurement time points to simulate intraoral conditions and maintain sample integrity. Color measurements were repeated after two weeks (T2) and one month (T3). Before each color measurement, samples were gently dried with sterile gauze to ensure consistent measurement conditions. Each measurement was taken three times per zone, and the mean of the three values was calculated to ensure measurement reliability. The color change (ΔE) was calculated between the baseline and each subsequent time point using the following formula:

$$\Delta E=\sqrt{{{\left(L2-L1\right)}^{2}+{\left(a2-a1\right)}^{2}+(b2-b1)}^{2}}$$

where L1, a1, and b1 represent baseline color values (T0) and L2, a2, and b2 represent the color values at subsequent time points. All values were tabulated for further statistical analysis.

### 2.6. Statistical Analysis

All collected data were analyzed using SPSS 24.0 (IBM, Armonk, NY, USA). A repeated-measures ANOVA was used to compare the color changes within and between groups over time. Post hoc pairwise comparisons were performed using a *t*-test, and the Bonferroni correction was used to identify significant differences among the groups. Statistical significance was set at *p* < 0.05.

## 3. Results

**Figure 2:**
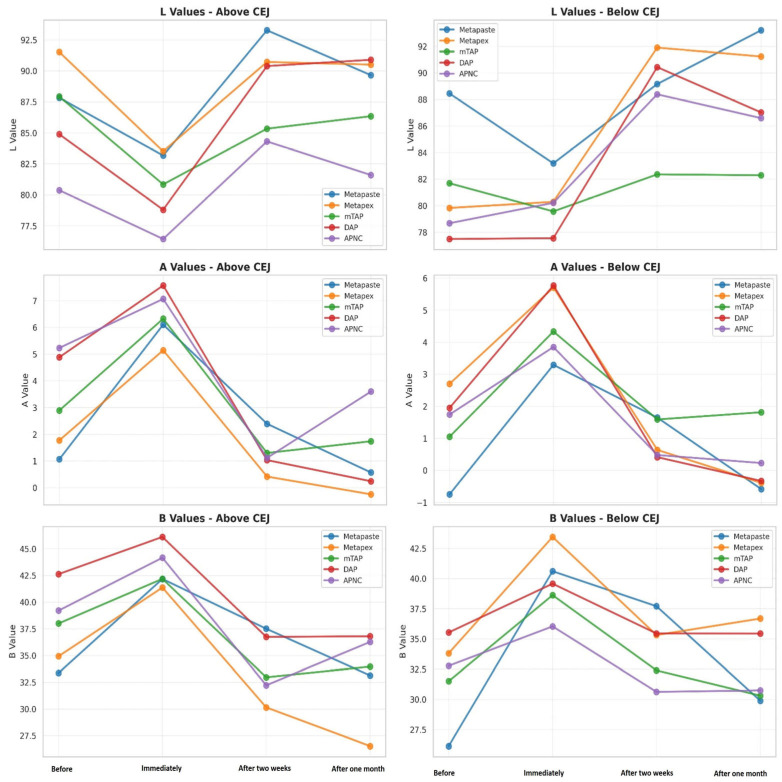
Change in the CIE parameter—L* luminescence, A* (red-green axis) and B* (yellow-blue axis) values of tested medicaments [Metapaste, Metapex, modified Triple Antibiotic Paste (mTAP), Double Antibiotic Paste (DAP), and antibiotic paste with Nano Chitosan (APNC)] at four time points above and below the cemento-enamel junction (CEJ).

**Figure 3:**
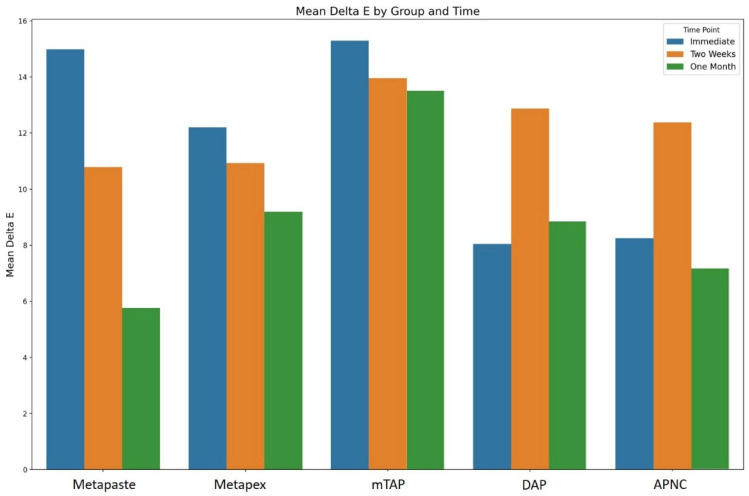
The overall degree of teeth discoloration after applying the tested medicaments [Metapaste, Metapex, modified Triple Antibiotic Paste (mTAP), Double Antibiotic Paste (DAP), and antibiotic paste with Nano Chitosan (APNC)] for three time points.

Tooth discoloration was observed across all groups and time points (Table 2). The color parameters (L*, a*, and b*) showed varying values with different observed time points and different zones (Figure 2). The overall degree of discoloration after applying the tested medicaments at three time points irrespective of zone is illustrated in Figure 3.

### 3.1. Discoloration Above CEJ (Figure 4)

After one month, the mTAP group exhibited the most pronounced discoloration, with an ΔE value of 8.45 ± 2.1, which was statistically greater than that of all other groups (*p* < 0.05). In contrast, Metapaste (ΔE = 3.59 ± 1.6) and APNC (ΔE = 4.15 ± 1.4) showed the lowest discoloration values, with no significant difference between them (*p* > 0.05). Intermediate levels of discoloration were observed in DAP (ΔE = 5.83 ± 2.7) and Metapex (ΔE = 5.48 ± 1.9).

Across time points, Metapaste, Metapex, and mTAP exhibited a gradual reduction in ΔE values, indicating a time-dependent stabilization or slight reversal of color change. Interestingly, both DAP and APNC displayed an increase in discoloration after two weeks (T2), followed by a decrease at the one-month mark (T3), suggesting a transient chromogenic effect. Metapaste, in particular, demonstrated a statistically significant reduction in discoloration over time (*p* < 0.01).

**Figure 4:**
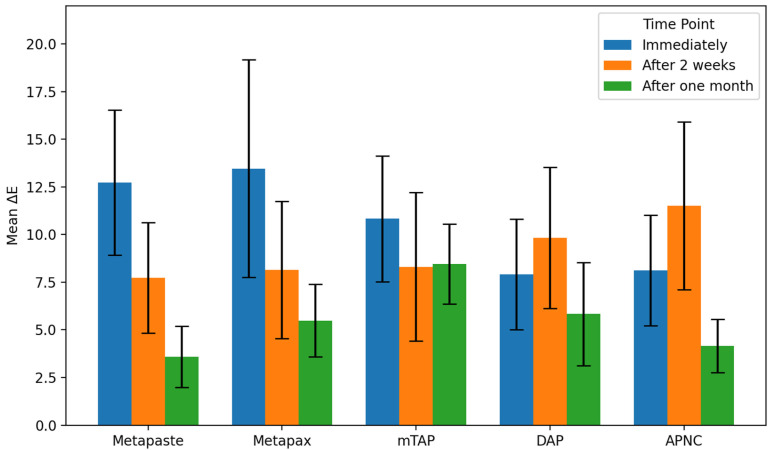
Degree of teeth discoloration above the cemento-enamel junction (CEJ) after applying the tested medicaments [Metapaste, Metapex, modified Triple Antibiotic Paste (mTAP), Double Antibiotic Paste (DAP), and antibiotic paste with Nano Chitosan (APNC)] for three time points.

### 3.2. Discoloration Below CEJ (Figure 5)

The mean values of ΔE below CEJ were generally higher than those observed above the CEJ across all groups. The mTAP group consistently exhibited the highest ΔE values at every time point, reaching 18.78 ± 4.4 after one month, which was significantly higher than all other medicaments (*p* < 0.05).

**Figure 5:**
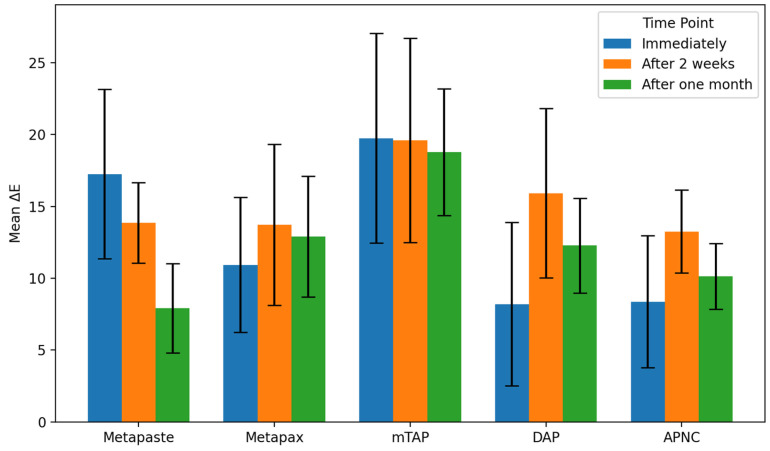
Degree of teeth discoloration below the cemento-enamel junction (CEJ) after applying the tested medicaments [Metapaste, Metapex, modified Triple Antibiotic Paste (mTAP), Double Antibiotic Paste (DAP), and antibiotic paste with Nano Chitosan (APNC)] for three time points.

**Figure 6:**
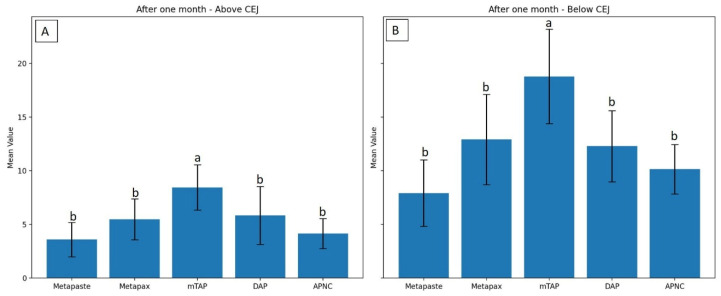
Degree of discoloration (ΔE) induced by tested medicaments after one month above the CEJ (**A**) and below the CEJ (**B**). Different letters indicate statistically significant differences between groups (*p* < 0.05).

Metapex also exhibited relatively high discoloration (ΔE = 12.91 ± 4.2) after one month; however, this was not statistically different from the values recorded for Metapaste, DAP, and APNC (*p* > 0.05). mTAP and Metapex did not show significant time-dependent changes in ΔE below the CEJ (*p* > 0.05). In contrast, Metapaste demonstrated a statistically significant reduction in ΔE values over time (*p* = 0.0001) despite its lower ΔE values overall. While DAP, Metapex, and APNC showed an increasing trend in discoloration over time, these changes were not statistically significant at the one-month evaluation (Figure 6 and Table 2).

## 4. Discussion

Tooth discoloration resulting from intracanal medicaments presents a significant esthetic challenge, particularly in cases where prolonged medicament application is essential for effective root canal disinfection, such as in the treatment of mature anterior teeth associated with persistent infections, where intracanal medicaments such as calcium hydroxide, bioactive glass, or antibiotic pastes are used for extended durations [13].

This study evaluated and compared the discoloration potential of five intracanal medicaments; Metapaste, Metapex, modified Triple Antibiotic Paste (mTAP), Double Antibiotic Paste (DAP), and an experimental antibiotic paste with Nano Chitosan (APNC) at two anatomical zones and across three time intervals. Based on the findings, significant differences were observed in discoloration between materials after one month, with variations depending on both location (above or below CEJ) and time. All three null hypotheses were therefore rejected. Previous studies have demonstrated that combinations of two or more antibiotics are more effective antimicrobials than single-drug applications [18,19]. The most recognized of these combinations is Triple Antibiotic Paste (TAP), developed by Hoshino et al. [4], and composed of ciprofloxacin, metronidazole, and minocycline. TAP is widely used in regenerative endodontics due to its broad-spectrum antimicrobial activity; however, concerns have been raised regarding its cytotoxicity to stem cells, particularly at high concentrations [7,20]. More critically, TAP is associated with significant tooth discoloration, primarily attributed to minocycline, a tetracycline derivative with a strong affinity for calcium ions and dentin collagen [21]. Although the exact mechanism is not fully clear, minocycline integrates into the dentin matrix, forming insoluble and pigmented complexes via oxidative pathways triggered by light or microbial activity, resulting in dark intrinsic discoloration that may penetrate deep into the dentin [22,23].

In the present study, minocycline was replaced by doxycycline in the mTAP formulation. Doxycycline is also a tetracycline derivative but is reported to have a lower staining potential especially when application is restricted below the CEJ [9,11]. Nevertheless, it can still chelate calcium and form complexes with dentin hydroxyapatite, resulting in yellow to brown stains [24]. Moreover, metronidazole was substituted with Amoxicillin + clavulanate in the mTAP to explore alternative combinations, addressing a gap in the literature regarding the chromogenic potential of non-minocycline antibiotic pastes [8]. Despite these modifications, mTAP exhibits the highest ΔE values in both zones above and below the CEJ over time, which aligns with earlier reports attributing color changes to the inclusion of tetracycline [8,23,25]. These findings confirmed the limitations of using tetracycline in the anterior esthetic zone [26].

Conversely, DAP, which excludes the tetracycline derivative, showed significantly less discoloration than mTAP. This came in agreement with previous findings in which the DAP was formulated by mixing equal portions of metronidazole and ciprofloxacin, and this mixture showed minimal discoloration and maintained adequate antimicrobial activity [8,27]. However, contrasting findings exist. For instance, a study involving a commercial DAP formulation (N-BI MIX) reported an ΔE value of 14.9 after one year, suggesting notable discoloration [28]. This discrepancy may be due to variations in vehicle composition, medicament concentration, or evaluation periods. While antibiotic pastes dominate many clinical and in vitro studies, recent guidelines increasingly advocate for the use of calcium hydroxide [12,29], due to its lower cytotoxicity toward apical papilla stem [7] and its reduced risk for tooth discoloration [11]. However, calcium hydroxide limitations include insufficient penetration of biofilms and lower effectiveness against resistant species like *Enterococcus faecalis* [30]. Prolonged calcium hydroxide exposure has also been linked to collagen degradation and demineralization of radicular dentin [31].

Metapaste is a water-based calcium hydroxide formulation with barium sulfate and propylene glycol. Metapaste showed a decreasing trend in Delta E values over time in both zones, suggesting that the color is becoming more stable and closer to baseline over time. These findings are partially consistent with prior studies showing that calcium hydroxide causes minimal or no tooth discoloration [8,11]. Although a negative control was not used in this study, Metapaste has previously served as a reference control in similar investigations [32]. Metapex, in contrast, is an oil-based paste calcium hydroxide formulation that includes iodoform, a chromogenic agent that can contribute to yellow discoloration. Its silicone oil base also slows calcium ion release compared to water-based pastes like Metapaste [33]. Both mTAP and Metapex showed stable ΔE values below the CEJ over time. This could be due to direct contact with dentin walls and enhanced paste penetration following smear layer removal with EDTA. Their viscosity may have restricted coronal spread, limiting discoloration above the CEJ.

The experimental APNC paste, composed of chitosan nanoparticles combined with ciprofloxacin and Amoxicillin + clavulanate, demonstrated significantly lower discoloration than mTAP and was comparable to Metapaste and DAP. Chitosan is a biocompatible polysaccharide derived from crustacean shells and is recognized for its antimicrobial, chelating, and biofilm-disruptive properties [14]. Its nanoparticulate form demonstrates effective antimicrobial properties and exhibits biocompatible and chelating properties [34]. After one month, APNC demonstrated significantly lower discoloration compared to mTAP and performed comparably to Metapaste and DAP. This is likely attributable to the absence of tetracycline antibiotics and the inherent chemical stability of chitosan nanoparticles, which do not interact with dentin in ways that lead to chromogenic by-products [35]. Its chelating ability may also prevent discoloration by limiting metal-ion-mediated oxidation reactions [36]. The interpretation of lightness (L*) values was complex and results were not clearly understood. A drop in L* immediately after application may be attributable to the extended effects of irrigation agents like NaOCl and EDTA, which alter dentin reflectivity. Variation in L values across materials and zones after one month could be attributed to differences in paste solubility, particle size, vehicles, and dentinal tubule diffusion capacity. Overall, discoloration was more pronounced below the CEJ in all groups, likely due to increased tubule density and depth of medicament penetration in this region, particularly after smear layer removal. Interestingly, discoloration levels in DAP, Metapex, and APNC increased slightly below the CEJ over time but plateaued by one month, suggesting a stabilization of chromogenic activity. This plateau may be beneficial for long-term esthetic outcomes.

Bovine teeth were used in this study due to their structural similarities to human dentin [37], availability, ease of collection, and proven validity for such types of studies [8,11]. Also, all teeth could be obtained on the same day and at a similar stage of tooth development. Their larger mesiodistal dimensions allowed for better adaptation of the spectrophotometer, enhancing measurement reliability. However, bovine dentin differs in mineral content and permeability from human teeth, and bovine root dentine has a significantly higher tubule density than human teeth, which may influence diffusion and discoloration dynamics [37]. Furthermore, natural human teeth present different baseline L*, a*, and b* values. Conflicting findings across similar previous studies may be attributed to differences in methodological models such as the use of various vehicles (e.g., distilled water, saline, macrogol ointment, and propylene glycol) [8], each of which may influence the diffusion characteristics of medicaments. Additionally, the observation periods varied among studies, ranging from one day to one year [11]. One major inconsistency lies in the determination of baseline time points for ΔE measurement. Some investigations considered the immediate post-application reading as the baseline [28,38], while others used the pre-application color measurements as the reference point (11, 23). This discrepancy affects the calculation and clinical interpretation of color change values. Furthermore, the method of sample preparation may also impact outcomes. In some studies, standardized cuboid enamel–dentin blocks with fixed dimensions were used to minimize variation [11,28], whereas other models utilized whole teeth, with access made through the apical aspect to preserve coronal structure [8].

In the current study, the procedure was performed from the coronal aspect to simulate a clinical scenario. Every effort was made to ensure that the tested materials were positioned below the CEJ. However, minor coronal contamination cannot be ruled out. One limitation of the current study was the absence of a comparison between observed ΔE values and known human perceptibility thresholds. One previous report suggests that ΔE values above 3.7 are visually detectable, while values above 5.5–6.8 warrant treatment intervention [39]. Another limitation was the lack of standardization in dentin wall thickness, which influences discoloration depth [38]. However, to overcome that, the teeth were randomly distributed among the groups. High concentrations of medicaments were used in this study to simulate the maximum discoloration scenarios within a one-month window. Future studies should investigate dose-depended tooth discoloration and cytotoxicity across a broader range of concentrations.

## 5. Conclusions

All tested intracanal medicaments induced some degree of tooth discoloration over time. The degree of discoloration was also influenced by the location (above vs. below CEJ), with most groups showing more pronounced discoloration below the CEJ. mTAP demonstrated the greatest potential for tooth discoloration both above and below the CEJ and should be used with caution where esthetics is a concern. Metapaste and APNC showed the least discoloration, making them preferable choices for minimizing esthetic impact. APNC, in particular, emerges as a promising alternative due to its favorable biocompatibility, antimicrobial profile, and low discoloration potential. Further, in vitro and in vivo investigations are needed to confirm the findings of the present study under different conditions and to assess the microbiological efficacy of mTAP and APNC compared to traditional medicaments.

## Figures and Tables

**Table 1:** Composition of the tested medicaments, percentage, and purpose of each component.

Medicament	Composition (Approximate Percentage)	Purpose
Metapaste	Calcium Hydroxide (40–50%)	Antibacterial and high pH.
	Barium Sulfate (20–30%)	Radiopacifier.
	Poly Propylene Glycol (20–30%)	Water-soluble vehicle.
	Inert Fillers (5–10%)	Stabilizers.
Metapex	Calcium Hydroxide (30–40%)	Antibacterial and high pH.
	Iodoform (30–40%)	Antiseptic.
	Silicone Oil (20–30%)	Oil-based vehicle.
	Inert Fillers (5–10%)	Stabilizers.
modified Triple Antibiotic Paste (mTAP)	Ciprofloxacin (25%)	Broad-spectrum antibiotic; effective against aerobic and anaerobic bacteria.
	Amoxicillin + Clavulanic Acid (25%)	A broad-spectrum covering aerobes and anaerobes is effective against resistant strains.
	Doxycycline (25%)	A semisynthetic tetracycline; targeting Gram-positive and Gram-negative bacteria.
	Distilled water (25%)	Vehicle.
Double Antibiotic Paste (DAP)	Ciprofloxacin (37%)	Broad-spectrum antibiotic; effective against aerobic and anaerobic bacteria.
	Metronidazole (37%)	Targets anaerobic bacteria.
	Distilled water (25%)	Vehicle.
Antibiotic paste with Nano Chitosan) (APNC)	Amoxicillin + Clavulanic Acid (25%)	Covering aerobes and anaerobes, effective against resistant strains.
	Ciprofloxacin (25%)	Effective against aerobic and anaerobic bacteria.
	Nano Chitosan (25%)	Antimicrobial biopolymer.
	Distilled water (25%)	Vehicle.

**Table 2:** Means and ±SD values of Delta E for five medicament groups at two zones at three time points.

Tested Medicaments	Zone	Time Point			
		Immediately	After 2 Weeks	After One Month	*p*-Value
Metapaste	Above CEJ	12.73 ^a^ ± 3.8	7.73 ^b^ ± 2.9	3.59 ^c^ ± 1.6	0.0001
	Below CEJ	17.25 ^a^ ± 5.9	13.85 ^a^ ± 2.8	7.92 ^b^ ± 3.1	0.0001
Metapax	Above CEJ	13.46 ^a^ ± 5.7	8.15 ^ab^ ± 3.6	5.48 ^b^ ± 1.9	0.0006
	Below CEJ	10.93 ^a^ ± 4.7	13.72 ^a^ ± 5.6	12.91 ^a^ ± 4.2	0.4319
mTAP	Above CEJ	10.83 ^a^ ± 3.3	8.31 ^a^ ± 3.9	8.45 ^a^ ± 2.1	0.1636
	Below CEJ	19.75 ^a^ ± 7.3	19.60 ^a^ ± 7.1	18.78 ^a^ ± 4.4	0.9356
DAP	Above CEJ	7.9 ^ab^ ± 2.9	9.83 ^a^ ± 3.7	5.83 ^b^ ± 2.7	0.0291
	Below CEJ	8.20 ^b^ ± 5.7	15.92 ^a^ ± 5.9	12.28 ^ab^ ± 3.3	0.0088
APNC	Above CEJ	8.12 ^a^ ± 2.9	11.50 ^a^ ± 4.4	4.15 ^b^ ± 1.4	0.0001
	Below CEJ	8.37 ^b^ ± 4.6	13.26 ^a^ ± 2.9	10.14 ^ab^ ± 2.3	0.0130

Means values with different superscript letters in the same raw are significantly different at *p* < 0.05.

## Data Availability

Data are available on request from the authors.

## Abbreviations

The following abbreviations are used in this manuscript:

CEJ	Cemento-enamel junction
mTAP	modified Triple Antibiotic Paste Directory
DAP	Double Antibiotic Paste
APNC	Antibiotic paste modified with Nano Chitosan
